# Lack of Galectin-3 Disrupts Thymus Homeostasis in Association to Increase of Local and Systemic Glucocorticoid Levels and Steroidogenic Machinery

**DOI:** 10.3389/fendo.2018.00365

**Published:** 2018-07-10

**Authors:** Ednéa Oliveira-de-Abreu, Danielle Silva-dos-Santos, Ailin Lepletier, Tiago D. P. Ramos, Rafaella Ferreira-Reis, Larissa Vasconcelos-Fontes, Mariana T. Ramos, Rafael C. Torres, Vinícius Cotta-de-Almeida, Vinícius de Frias Carvalho, Déa M. S. Villa-Verde

**Affiliations:** ^1^Laboratory on Thymus Research, Oswaldo Cruz Institute, Oswaldo Cruz Foundation, Rio de Janeiro, Brazil; ^2^Laboratory of Inflammation, Oswaldo Cruz Institute, Oswaldo Cruz Foundation, Rio de Janeiro, Brazil; ^3^National Institute of Science and Technology on Neuroimmunomodulation (INCT-NIM), Rio de Janeiro, Brazil

**Keywords:** galectin-3, thymus, thymocytes, proliferation, cell death, glucocorticoid, steroidogenic machinery

## Abstract

Maintenance of thymus homeostasis is a delicate interplay involving hormones, neurotransmitters and local microenvironmental proteins, as well as saccharides, acting on both thymocytes and stromal cells. Disturbances in these interactions may lead to alterations on thymocyte development. We previously showed that galectin-3, a β-galactoside-binding protein, is constitutively expressed in the thymus, interacting with extracellular matrix glycoproteins and acting as a de-adhesion molecule, thus modulating thymocyte-stromal cell interactions. In this work, we aimed to investigate the participation of galectin-3 in the maintenance of thymus homeostasis, including hormonal-mediated circuits. For that, we used genetically engineered galectin-3-deficient mice. We observed that the thymus of galectin-3-deficient mice was reduced in mass and cellularity compared to wild-type controls; however, the proportions of different thymocyte subpopulations defined by CD4/CD8 expression were not changed. Considering the CD4^−^CD8^−^ double-negative (DN) subpopulation, an accumulation of the most immature (DN1) stage was observed. Additionally, the proliferative capacity of thymocytes was decreased in all thymocyte subsets, whereas the percentage of apoptosis was increased, especially in the CD4^+^CD8^+^ double-positive thymocytes. As glucocorticoid hormones are known to be involved in thymus homeostasis, we evaluated serum and intrathymic corticosterone levels by radioimmunoassay, and the expression of steroidogenic machinery using real-time PCR. We detected a significant increase in corticosterone levels in both serum and thymus samples of galectin-3-deficient mice, as compared to age-matched controls. This was paralleled by an increase of gene transcription of the steroidogenic enzymes, steroidogenic acute regulatory protein (*Star*) and *Cyp11b1* in thymus, 11β-Hydroxysteroid Dehydrogenase (*Hsd11b1)* in the adrenal, and *Cyp11a1* in both glands. In conclusion, our findings show that the absence of galectin-3 subverts mouse thymus homeostasis by a mechanism likely associated to intrathymic and systemic stress-related endocrine circuitries, affecting thymocyte number, proliferation and apoptosis.

## Introduction

Galectins are a family of 15 β-galactoside-binding lectins, containing at least one conserved carbohydrate-recognition domain, which can be found in the nucleus, cytosol, and bound to cell membrane glycoconjugates or to extracellular matrix glycocomponents (1). Depending to their location into the cell, galectins can influence cell proliferation, adhesion, migration, signaling, differentiation, and apoptosis (1, 2). They were also shown to modulate immune functions in health and disease (3, 4). We showed that galectin-3 is constitutively expressed by epithelial and phagocytic cells in both thymic cortex and medulla. Galectin-3 interacts with glycoconjugates on thymocyte surface and extracellular matrix glycoproteins acting as a de-adhesion molecule, thus modulating thymocyte-stromal cell interactions (5). Furthermore, we also noted that galectin-3 accumulates in the thymus of *Trypanosoma cruzi* infected mice, being related to increased thymocyte death and exit to the periphery, and consequent thymus atrophy (6).

Thymus involution is a physiological phenomenon of the organ, related to aging, leading to progressive alterations in the thymus microenvironment, with loss of thymus mass, thymic epithelial cell (TEC) number and function, resulting in a decrease in thymopoiesis. In consequence, a decrease in the immune function is observed in the elderly, with less resistance to infections, autoimmune diseases and cancer (7, 8). On the other hand, acute thymic involution is related to pathological conditions, such as metabolic and infectious diseases (9, 10).

In addition, activation of the hypothalamus–pituitary–adrenal axis induced by stress or some diseases, including diabetes and Chagas disease, was shown to cause severe atrophy of the thymus (11, 12). Glucocorticoids decrease proliferation and increase apoptosis of immature CD4^+^CD8^+^ double-positive (DP) thymocytes, inducing a strong atrophy in the thymus (13, 14). These actions of glucocorticoids on thymus are related to endocrine and paracrine actions of this hormone, since thymus presents the steroidogenic machinery, including StAR, 11β-HSD1, and 11β-HSD2, and is capable to produce glucocorticoids (15, 16).

Considering the multifunctional role of galectin-3 as a regulator of cell adhesion, migration, proliferation, signaling, differentiation and apoptosis, our hypothesis is that galectin-3 is a key player to maintain thymus homeostasis. Here, we undertook this study to evaluate the role of galectin-3 on thymus homeostasis in association with its effects on local and systemic production of glucocorticoids.

## Materials and methods

### Mice

Male BALB/c wild type (WT) and galectin-3 deficient mice (4–6 week old) were obtained from the Oswaldo Cruz Foundation animal facilities, Rio de Janeiro, Brazil. Galectin-3 deficient mice (Gal-3^−/−^) were generated by backcrossing with their BALB/c littermates for 9 generations (17). Mice were housed in groups of three in a temperature-, humidity-, and light-controlled (12 h light: 12 h darkness cycle) colony room. Mice were given *ad libitum* access to food and water. All protocols for the use and care of animals were approved by the Ethics Committee for the Use of Experimental Animals of the Oswaldo Cruz Foundation, under licenses number L-024/09 and L-004/2014.

### Analysis of T cell subpopulations

Individual thymuses were minced, resuspended in phosphate buffered saline solution (PBS) (Sigma Aldrich, St Louis, MO, USA) with 5% Fetal Calf Serum (FCS) (Cultilab, Campinas, SP, Brazil) and counted in Neubauer chamber in the presence of Trypan Blue (Sigma Aldrich) for evaluation of cell viability. Trypan Blue evaluation of cell viability in fresh thymocytes showed that about 95 and 85% of cells were alive in the thymus of WT and Gal-3^−/−^ mice, respectively. The phenotype of the main thymocyte subpopulations was evaluated by Flow Cytometry with the use of monoclonal antibodies to mouse CD4, CD8, CD44, and CD25 conjugated to different fluorochromes (BD, San Diego, CA, USA). Control isotype immunoglobulins conjugated to correspondent fluorochromes were used for negative staining determination (BD). Briefly, 10^6^ thymocytes were incubated for 15 min with 2 μL of normal mouse serum for blockage of unspecific binding, and subsequently with 10 μL of different antibody combinations for 30 min. Cells were then washed in PBS, fixed with 1% formaldehyde and analyzed by Flow Cytometry in a FACSCanto II equipped with the FACSDiva Software (BD). Data were analyzed with the Summit 4.3 Software (Dako Cytomation,Carpinteria, CA, USA).

### Evaluation of thymocyte proliferation

For evaluation of spontaneous thymocyte proliferation, thymocytes were incubated for 3 h in RPMI medium (Sigma Aldrich) with 10% FCS containing 60 μM bromodeoxyuridine (BrdU) (Sigma Aldrich). Cells were then incubated with anti-CD4, anti-CD8, anti-CD44, anti-CD25 monoclonal antibodies (BD) conjugated to different fluorochromes. After washings, cells were permeabilized using the kit BD Cytofix/Cytoperm™ (BD). Subsequently, BrdU incorporated to cell DNA was exposed by treatment of cells with 100U DNAse I (Roche, Mannheim, BW, Germany) for 40 min at room temperature. Cells were then washed twice for 5 min at 450 × g, and subsequently incubated with FITC conjugated anti-BrdU (eBioscience, Inc., San Diego, CA, USA). Samples were acquired in a FACSCanto II device (BD) and analyzed with Summit 4.3 Software (Dako Cytomation).

### Measurement of thymocyte apoptosis

For evaluation of cell death, thymocytes were first stained for surface molecules CD4 and CD8 conjugated to different fluorochromes (BD), washed, suspended in Annexin V buffer and treated for 10 min with 1 μL Annexin V conjugated to fluorescein isothiocyanate (FITC) (BD) according to the manufacturer's recommendations. Cells were immediately analyzed by Flow Cytometry using a FACSCanto II device (BD) equipped with the FACSDiva Software (BD).

### Immunofluorescence

The evaluation of thymic epithelial compartment was performed by immunofluorescence. Thymuses (5 animals/group) were removed and frozen in Tissue Tek (Optimal Cutting Temperature Compound, Sakura Finetek, Torrance, CA, USA). Slices of 5 μm-thick thymic sections were obtained in cryostat (Leica CM 1850 - Leica Microsystems Inc.; Buffalo Grove, IL, USA) and fixed in cold acetone for 5 min. Tissue sections were then incubated with 2.5% BSA in PBS for 1 h and subsequently subjected to the indirect immunofluorescence technique for immunolabeling with pan-cytokeratin antibody (Dako), or unrelated control IgG (Molecular Probes, Carlsbad, CA, USA) for 1 h at room temperature. Sections were then washed three times in PBS and incubated for 45 min with the secondary anti-rabbit antibody conjugated to Alexa Fluor 546 (Molecular Probes). Sections were washed again three times in PBS and mounted with Fluoroshield containing 4′,6-diamidino-2-phenylindole - DAPI (Sigma Aldrich) for nuclear staining. Samples were analyzed using a Carl Zeiss Axio Imager Upright Microscope (Zeiss, Oberkochen, BW, Germany).

### Histology

Thymus histological analysis was performed by Hematoxylin & Eosin technique. Thymuses (3 animals/group) were fixed in buffered 10% formalin (Millonig buffer) for 48 h. Paraffin-embedded 5-μm sections were mounted on glass slides. The sections were deparaffinized with xylene, and rehydrated by a graded series of ethanol washes. Sections were then left in running water for 1 min, stained with Hematoxylin for 10 min, washed in running water for 1 min, and incubated in Eosin solution for 3 min (Sigma, Aldrich). Photos were taken using the Leica DM 2500 microscope.

### Evaluation of corticosterone levels

Serum and thymus samples were obtained simultaneously from WT and Gal-3^−/−^ mice.

Animals were euthanized in a CO2 chamber, during the nadir (08:00 h) of the circadian rhythm as described previously (18), and the blood was immediately collected from the abdominal aorta. After blood coagulation, individual sera was collected and stored at −20°C until use. Thymus samples were obtained and kept at a −20°C until use. After thawing, the thymuses were suspended in 150 μl PBS and then triturated in tissue homogenizer. The homogenates were centrifuged at 10,000 × g for 15 min at 4°C. Serum and thymus corticosterone levels were detected by radioimmunoassay (RIA) following manufacturer's guidelines (MP Biomedicals, Solon, OH, USA). Final intrathymic corticosterone levels were represented by the ratio of hormone concentration in supernatants and thymus mass.

### Gene expression of steroidogenic enzymes in adrenals and thymuses

Thymus and adrenal total RNA from WT and Gal-3^−/−^ animals were obtained using the RNeasy Micro Kit (Qiagen, Valencia, CA, USA). The quantification was performed in the spectrophotometer NanoDrop 1000 (Thermo Ficher Scientific, Waltham, MA, USA). For the synthesis of cDNA, equivalent samples were used in 1 μg of RNA, using SuperScript III First-Strand Synthesis System (Invitrogen, Carlsbad, CA, USA), in the presence of a random primer, according to the manufacturer's recommendation. For the analysis of gene expression by real-time PCR, 100 ng of cDNA samples were diluted in Power SYBR Green PCR Master Mix (Applied Biosystems, Carlsbad, CA, USA) in the Step One Plus system (Applied Biosystems). The PCR method was performed at 95°C for 10 min followed by 40 cycles at 95°C for 15 s, 60°C for 1 min. The specificity of reaction products was verified through the dissociation curve. The data were analyzed by ABI Prism SDS v1.3.1 software. All primers were designed using the Primer Express 3.0 specific program for 7500 FAST Real Time PCR System. cDNA was amplified using specific murine primer sequences described in Table 1. After 40 cycles of amplification, expression of cytochrome P450, family 11, subfamily a, polypeptide 1 (*Cyp11a1*), *Cyp11b1*, steroidogenic acute regulatory protein (*Star*), and hydroxysteroid 11-beta dehydrogenase 1 (*Hsd11b1*) was assessed by comparing the expression of each to the normalizer constitutive reference transcript *Rpl-13a* (ribosomal protein L13A), using the Ct method as previously described (2^−dCt^ × 1,000) (19), subsequent to the following primer efficiency analysis. Each experiment was run in triplicate with different cDNA preparations from the same mice.

**Table 1:** Sequence of specific murine primers used for real time qPCR.

**Gene (primer)**	**Sequence**
***Star***	
Forward	5′-TCACTTGGCTGCTCAGTATTGAC-3′
Reverse	5′-GCGATAGGACCTGGTTGATGA-3
***Cyp11a1***	
Forward	5′-GACCTGGAAGGACCATGCA-3′
Reverse	5′-TGGGTGTACTCATCAGCTTTATTGA-3
***Cyp11b1***	
Forward	5′-TCAGTCCAGTGTGTTCAACTATACCA-3′
Reverse	5′- GCCGCTCCCCAAAAAGA-3′
***Gapdh***	
Forward	5′-CCATCACCATCTTCCAGGAG-3′
Reverse	5′-GCATGGACTGTGGTCATGAG-3′
***Hsd11d1***	
Forward	5′-TGGTGCTCTTCCTGGCCTACT-3′
Reverse	5′-CTGGCCCCAGTGACAATCA-3′
***Rpl13***	
Forward	5′-CCAAGCAGGTACTTCTGGGCCGGAA-3′
Reverse	5′-CAGTGCGCCAGAAAATGCGGC-3′

### Galectin-3 inhibition experiments

In order to investigate the possibility that the lack of galectin-3 is related to thymocyte death observed in our Gal-3^−/−^ mice, we performed *in vitro* experiments using GCS-100, a modified citrus pectin described to act as galectin-3 inhibitor (kindly donated by Dr. S. Patel, La Jolla Pharmaceutical Company, San Diego, CA). Briefly, 10^6^ thymocytes of 3-5 WT mice were cultured in RPMI 1640 medium supplemented with 10% fetal calf serum, 2 mM L-glutamine, 1 mM sodium pyruvate, 55 μM 2-mercaptoethanol (Gibco, Grand Island, NY), 100 U/mL penicillin, 0.1 mg/mL streptomycin and 0.25 μg/mL amphotericin B (Sigma Aldrich) in the presence of GCS-100, in the concentrations of 50–800 μg/mL, for 24 h at 37°C in a humidified atmosphere containing 5% CO_2_. For comparison we included in the experiment thymocytes treated with dexamethasone (0.01 μM)_._ Cells were subsequently incubated with antibodies to CD3, CD4 and CD8 conjugated to different fluorochromes (BD), washed, suspended in Annexin V buffer and treated for 10 min with Annexin-V and 7-AAD for viability evaluation. Cells were immediately analyzed by Flow Cytometry using a FACSCanto II device (BD) equipped with the FACSDiva Software (BD).

### Statistical analysis

Data were evaluated to ensure normal distribution and were statistically analyzed by unpaired *t*-test or ANOVA using the Tukey's multiple comparison test. Data are shown as individual values and median or mean ± standard error (used for real time PCR analysis). Tests were performed using GraphPad Prism 5.0 software (Graphpad Software, San Diego, CA, USA).

## Results

### Lack of galectin-3 induces thymus atrophy with microenvironmental alterations

**Figure 1:**
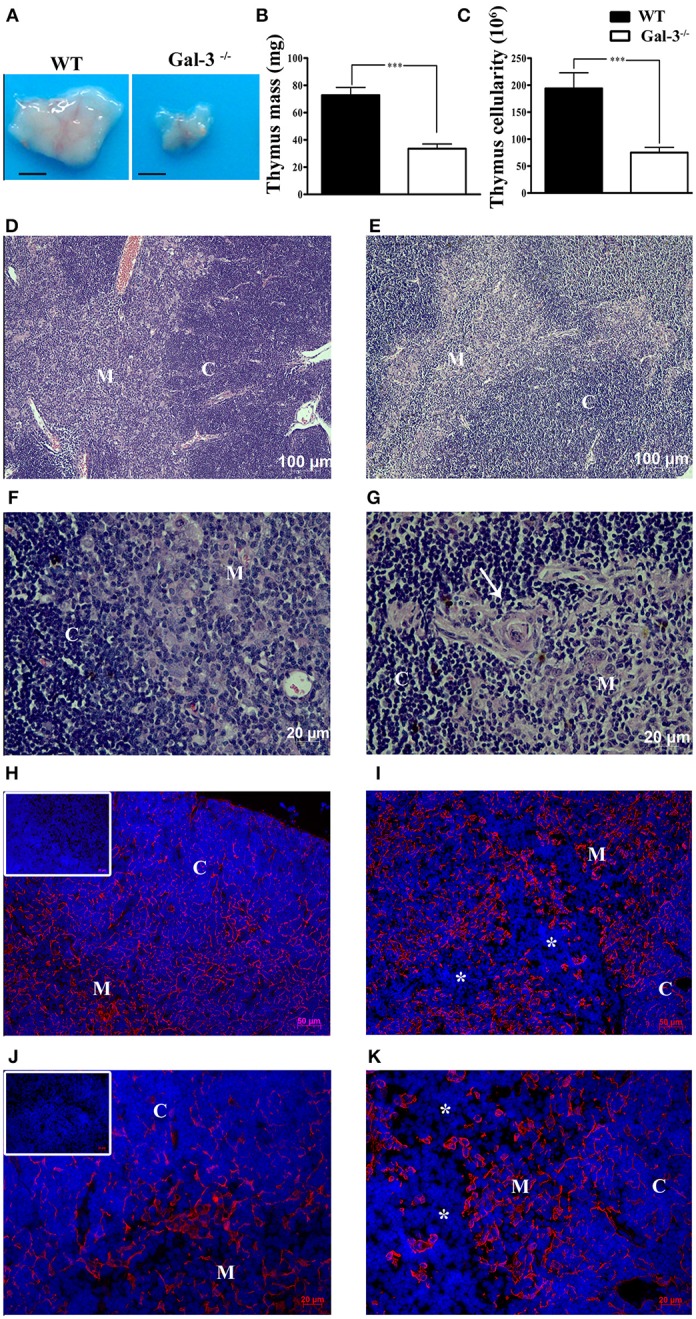
Thymus atrophy and microenvironmental alterations in the absence of galectin-3. **(A)** Shows comparative thymus pictures of WT and Gal-3^−/−^ mice. Thymus mass **(B)** and cellularity **(C)** of Gal-3^−/−^ mice are shown in comparison to WT control mice. Hematoxylin & Eosin stained sections of thymus of WT **(D,F)** and Gal-3^−/−^ **(E,G)** mice. Presence of concentric Hassall body-like structures is shown in Gal-3^−/−^ thymus (white arrow in **G**) but not in WT mice **(F)**. Immunofluorescence staining with anti-pan-cytokeratin is shown in the thymus of WT **(H,J)** and Gal-3^−/−^ **(I,K)** mice. Inserts in **(H,J)** show negative controls. Asterisks in **(I**,**K)** denote DAPI stained TEC-free regions. Blue staining in panels: DAPI, used to show cell nuclei. Images are representative of 5 animals/group. Bar in **(A)**: 0.25 cm. C, Cortex; M, Medulla.

We initially observed that galectin-3 deficient mice (Gal-3^−/−^) have a significant thymus atrophy with lower mass and cellularity compared to wild type (WT) BALB/c mice (Figures 1A–C). It is important to note that no significant differences were observed considering body mass of both strains of mice. Once the maintenance of adequate thymic architecture is fundamental to thymocyte differentiation, thymus atrophy is frequently accompanied by morphological tissue alterations. So, we evaluated thymus microscopic structure in Gal-3^−/−^ mice using Hematoxylin & Eosin staining. We observed that thymus cortex and medulla are preserved in both WT and Gal-3^−/−^ mice (Figures 1D,**E**). However we noticed the presence of concentric structures formed by TEC, similar to Hassall bodies, in the thymic medullary region of Gal-3^−/−^ mice (Figure 1G), that are not seen in WT thymuses (Figure 1F). We also evaluated the status of the epithelial component of thymic microenvironment by staining thymus sections with anti-pan-cytokeratin antibody. Immunofluorescence data showed a deep disorganization of the thymic epithelial network, with visible TEC-free regions in the thymus of Gal-3^−/−^ mice (Figures 1I,K) that were not observed in WT animals (Figures 1H,J).

### Lack of galectin-3 modulates thymocyte differentiation

**Figure 2:**
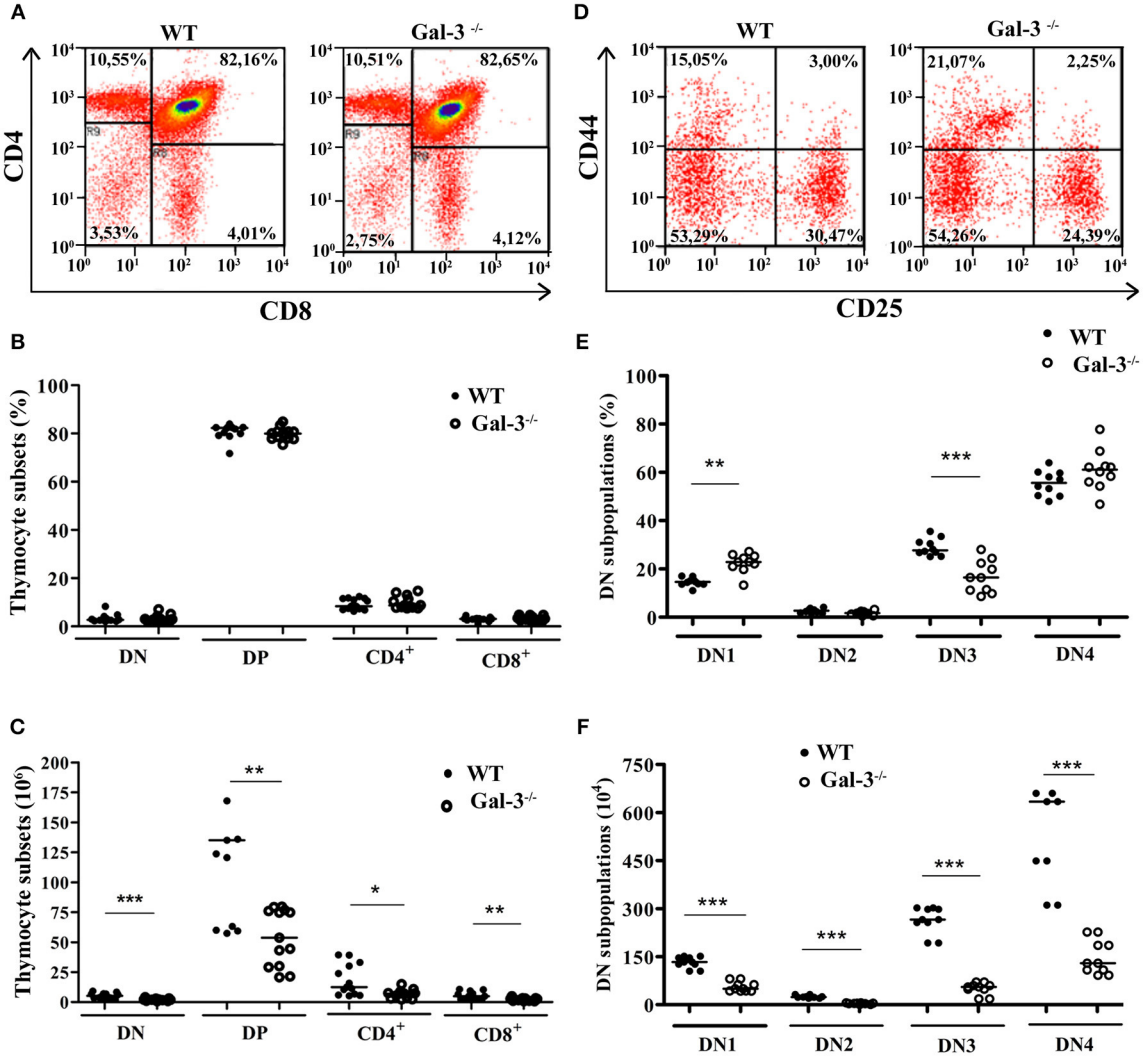
Modulation of thymocyte differentiation in the absence of galectin-3. **(A)** Shows representative dot plots obtained after CD4/CD8 staining of WT and Gal-3^−/−^ thymocytes. **(B**,**C)** Respectively show percentage and absolute numbers of thymocyte subpopulations defined by CD4/CD8 staining of WT and Gal-3^−/−^ mice. Data are representative of 14 animals/group. **(D)** Shows representative dot plots for CD44/CD25 staining of WT and Gal-3^−/−^ thymocytes. **(E**,**F)** Respectively show percentage and absolute numbers of DN thymocyte subpopulations defined by CD44/CD25 staining of WT and Gal-3^−/−^ mice. Data are representative of 10 animals/group. *p < 0.05; **p < 0.01; ***p < 0.001.

In order to verify if galectin-3 interferes with thymocyte differentiation, we analyzed thymocyte phenotype by flow cytometry using the membrane markers CD4, CD8, CD25 and CD44. We did not find changes in the percentage of thymocyte subpopulations defined by CD4 and CD8 when we compared the two strains of mice (Figures 2A,B). However, considering absolute cell numbers, Gal-3^−/−^ mice showed a significant decrease in all thymocyte subpopulations (Figure 2C).

The most immature thymocytes are DN for CD4 and CD8, and these cells can be further subdivided in four subsets considering the expression of CD25 and CD44 on their cell membrane. The homeostasis of DN thymocytes is crucial for thymocyte development, as during this stage extensive proliferation, TCR rearrangement and commitment to the αβ or γδ T cell lineages take place. DN1 cells, the most immature DN subpopulation, express CD44 on their cell membrane, but not CD25. Thymocytes sequentially express both CD44 and CD25 (DN2 cells), then lose the expression of CD44 and express only CD25 (DN3 cells), and ultimately are negative for both CD44 and CD25 (DN4 cells; also known as pre-DP) (20). Analysis of DN thymocyte subpopulations in the Gal-3^−/−^ mice showed a significant increase in the percentage of DN1 thymocytes and a decrease in cells in the DN3 stage compared to control WT animals, without alterations in the percentage of DN2 and DN4 cells (Figures 2D,E). In absolute numbers, Gal-3^−/−^ mice showed a decrease in all DN subpopulations (Figure 2F).

### Lack of galectin 3 interferes with thymocyte proliferation and death

**Figure 3:**
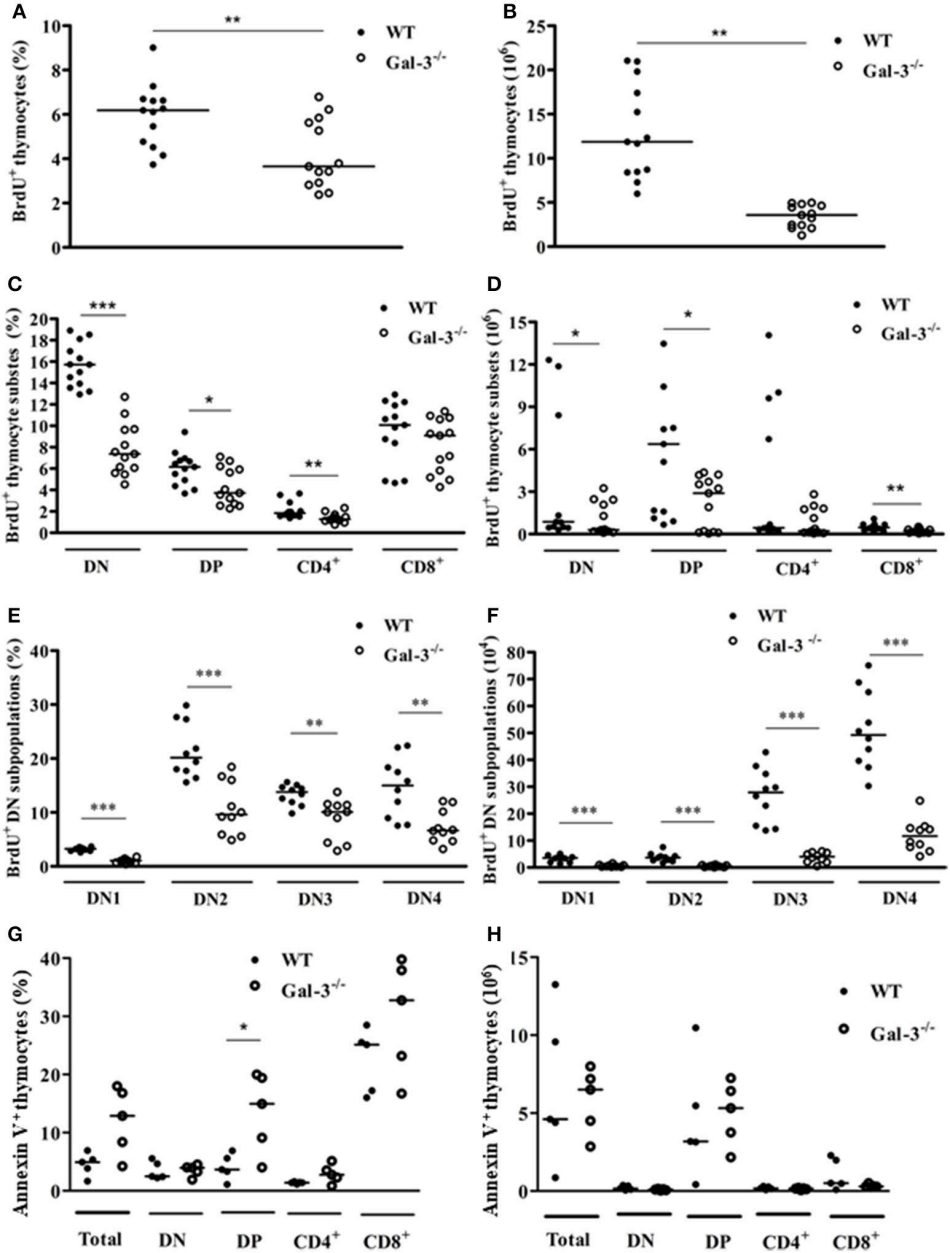
Changes in thymocyte proliferation and death in the absence of galectin-3. BrdU incorporation by total thymocytes of WT and Gal-3^−/−^ mice is shown in percentage **(A)** and absolute cell numbers **(B)**. **(C**,**D)** Respectively show BrdU incorporation by CD4/CD8-defined thymocyte subpopulations in percentage and absolute numbers. **(E**,**F)** Respectively show BrdU incorporation by DN thymocyte subpopulations in percentage and absolute numbers. Data are representative of 10 animals/group. **(G**,**H)** Respectively show percentage and absolute numbers of Annexin V^+^ cells in total and CD4/CD8-defined thymocyte subpopulations. Data are representative of 5 animals/group. *p < 0.05; **p < 0.01; ***p < 0.001.

Proliferation and apoptosis are important events for thymocyte development and maintenance of thymus cellularity. We evaluated if the decrease in thymus mass and cellularity observed in Gal-3^−/−^ mice was related to changes in the rates of both phenomena. Spontaneous thymocyte proliferation, evaluated after 3-h incubation with the thymidine analog BrdU, was decreased both in percentages and absolute numbers in total Gal-3^−/−^ thymocytes when compared to WT (Figures 3A,B). Percentages of spontaneous proliferation were decreased in DN, DP and CD4^+^ subpopulations (Figure 3C). However, considering absolute numbers DN, DP and CD8^+^, but not CD4^+^ cells showed decreased proliferation rate (Figure 3D). We finally evaluated the levels of spontaneous proliferation in DN subsets of thymocytes and observed a decrease in all DN subpopulations, from DN1 to DN4, both in percentage and absolute numbers (Figures 3E,F).

The results of cell death analysis, evaluated after labeling cells with Annexin V, showed statistically significant increase in the percentage of Annexin V^+^ DP thymocytes of Gal-3^−/−^ mice, compared to WT animals (Figure 3G). No differences were observed in absolute numbers (Figure 3H).

### Lack of galectin-3 increases serum and intrathymic corticosterone levels

**Figure 4:**
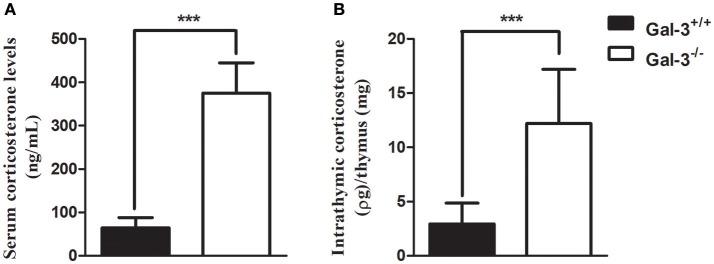
Increase in serum and intrathymic corticosterone levels in the absence of galectin-3. Radioimmune assay analysis of serum **(A)** and intrathymic **(B)** corticosterone levels of WT and Gal-3^−/−^ mice. Final intrathymic corticosterone levels were represented by the ratio of hormone concentration in supernatants and thymus mass. Data are representative of 10 animals/group. ***p < 0.001.

**Figure 5:**
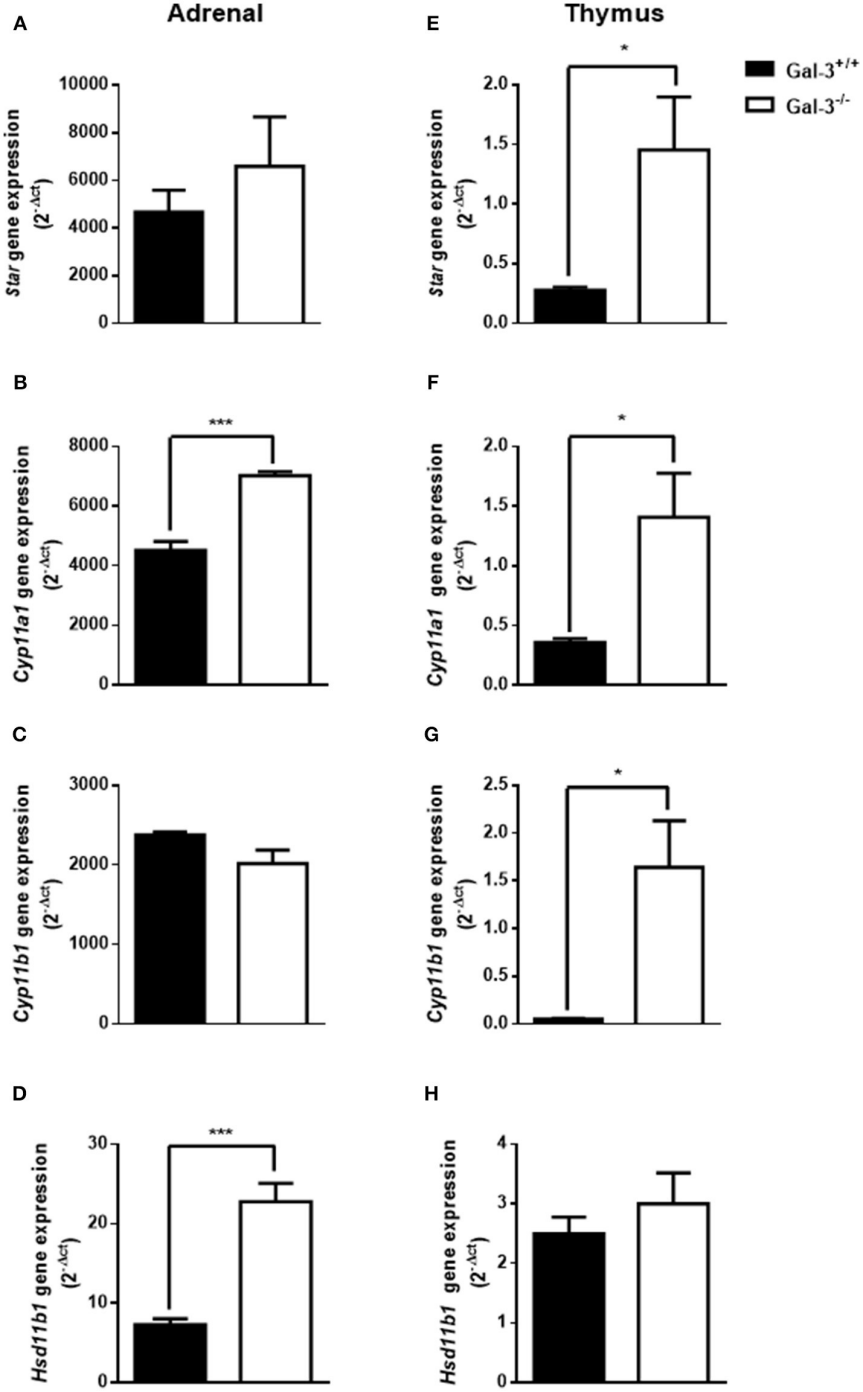
Changes of steroidogenic machinery in the absence of galectin-3. Gene expression of steroidogenic enzymes *Star* **(A,E)**, *Cyp11a1* **(B,F)**, *Cyp11b1***(C,G)**, and *Hsd11b1* **(D,H)** in adrenals and thymuses of WT and Gal-3^−/−^ mice was measured by qPCR. The values were normalized to the constitutive reference transcript *Rpl-13a*. Values are represented as (2-dct) of gene expression are shown as mean ± standard error. Each experiment was run in triplicate with different cDNA preparations from the same mice. Data are representative of 4 animals/group. *p < 0.05; ***p < 0.001.

Considering the participation of glucocorticoids on thymus involution, we initially evaluated the levels of corticosterone in the serum and thymus of both strains of mice. We detected high corticosterone levels in both serum and thymus of Gal-3^−/−^ mice compared to WT animals (Figure 4). In adrenal glands, Gal-3^−/−^ mice presented an increase in the expression of steroidogenic enzyme genes *Cyp11a1* (Figure 5B) and *Hsd11b1* (Figure 5D), but did not alter the gene expression of *Star* (Figure 5A) and *Cyp11b1* (Figure 5C), while in the thymus of Gal-3^−/−^ mice we noticed higher expression of *Star* (Figure 5E), *Cyp11a1* (Figure 5F), and *Cyp11b1* (Figure 5G), and no difference in *Hsd11b1* (Figure 5H) gene expression.

### Inhibition of galectin-3 in WT mice increases thymocyte apoptosis

**Figure 6:**
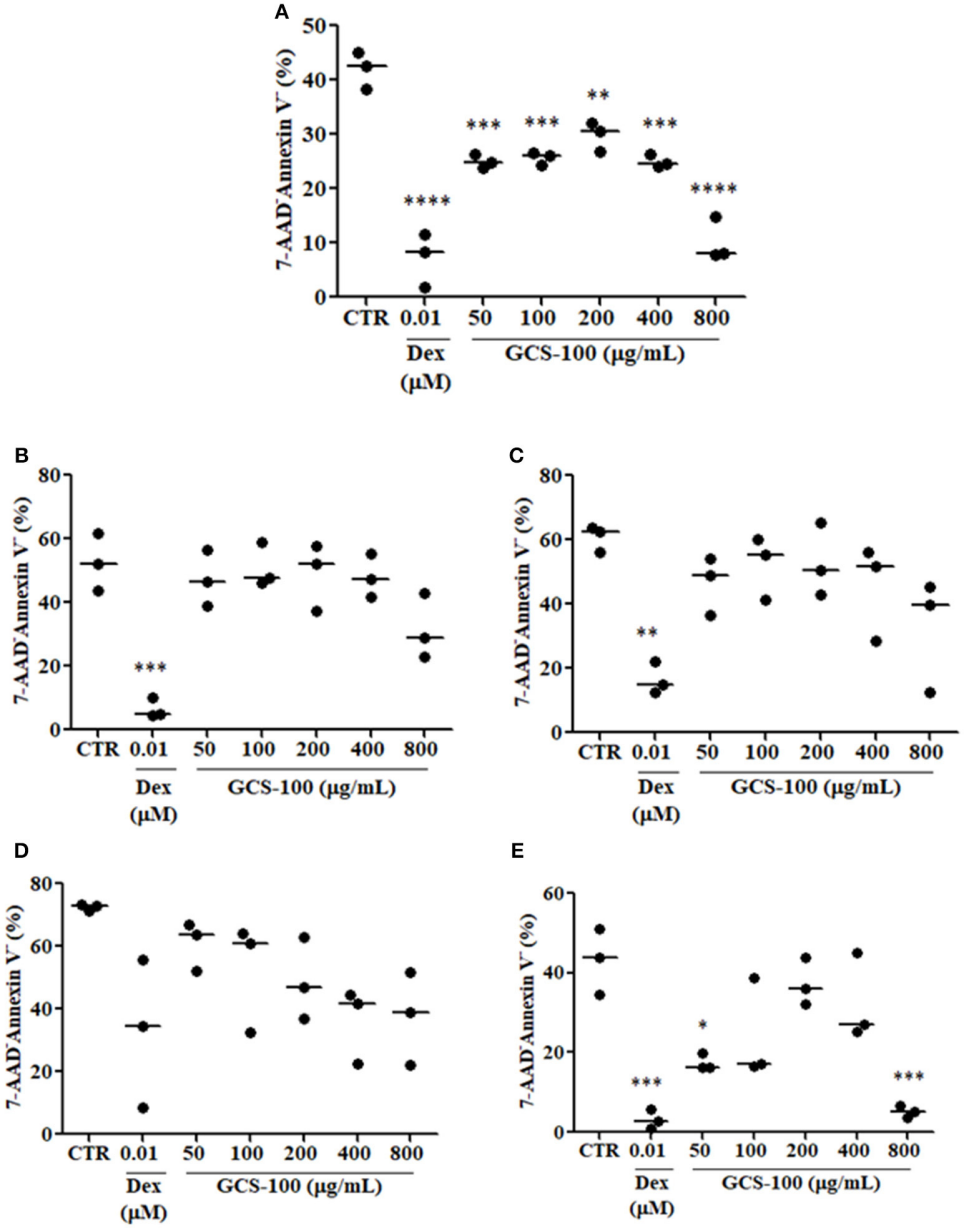
Inhibition of galectin-3 increases thymocyte apoptosis. **(A)** Shows % of living cells (7-AAD^−^/Annexin V^−^) in total thymocytes treated for 24 h with different concentrations of GCS-100 (50–800 μg/mL) or dexamethasone (0.01 μM). **(B**–**E)** Respectively show % of living cells (7-AAD^−^/Annexin V^−^) in CD4SP, CD8SP, DN/CD3^−^ and DP thymocyte subpopulations submitted to the same treatments. Data show one representative of two independent experiments with 3 animals/group, with similar results. *p < 0.05; **p < 0.01; ***p < 0.001; ****p < 0.0001.

To determine in which extent the increased thymocyte apoptosis observed in Gal-3^−/−^ mice was due to the primary lack of galectin-3 or to the high corticosterone levels observed in these animals, we treated thymocytes obtained from WT mice with different concentrations of GCS-100, a modified citrus pectin described to function as a galectin-3 inhibitor (21, 22), or dexamethasone *in vitro* for 24 h. Our results showed that thymocytes treated with different concentrations of GCS-100 for 24 h were more susceptible to apoptosis than untreated cells, as shown by the staining with 7-AAD/Annexin V (Figure 6). Moreover, DP thymocytes were the most susceptible cells comparing different thymocyte subpopulations. As expected, dexamethasone-treated thymocytes were induced to apoptosis, mainly DP cells (Figure 6E).

## Discussion

Thymocyte differentiation from bone marrow-derived precursors is dependent on their interactions with the thymic microenvironment, composed of stromal cells, namely TEC, fibroblasts, macrophages, dendritic cells; extracellular matrix (ECM) molecules, represented by fibronectin, laminin, type IV collagen; and soluble proteins, such as cytokines, chemokines, galectins, and with the neuro and endocrine systems (12, 23). In the present study we showed that the lack of galectin-3 consistently affected thymus homeostasis in association to local and systemic glucocorticoid production. Initially, we noticed that thymus mass and cellularity were significantly decreased in Gal-3^−/−^ mice compared to WT, presenting also alterations in the epithelial component of the thymic microenvironment, with regions without TEC in the thymus. These alterations in the structure of thymus epithelial network observed in Gal-3^−/−^ mice could, at least partly, explain the reduction of cellularity in this organ, once we previously demonstrated that galectin-3 is expressed by epithelial cells of both thymus cortex and medulla, playing a de-adhesive role by modulating thymocyte interactions with stromal cells and ECM components (5). Furthermore, the present data suggest that galectin-3 is not only important for the interactions of thymocytes with the thymic stroma, but also to the maintenance of thymic architecture and thymocyte homeostasis.

Next, we showed that the absolute numbers of different thymocyte subsets, defined by CD4 and CD8 molecules, were affected in Gal-3^−/−^ mice. The lack of galectin-3 seems to affect all thymocyte subpopulations, impacting the organ as a whole. Interestingly, considering DN thymocytes, the most immature subset in which important events of thymocyte differentiation occur, we observed an accumulation of DN1 and a decrease in DN3 cells, whereas in absolute numbers all DN subpopulations were decreased. In fact, we detected that thymocytes of Gal-3^−/−^ mice proliferate significantly less than those of WT, and this reduction was importantly noted in DN thymocytes. These alterations may affect all thymocyte development, leading to decreased thymus mass and cellularity, as in the DN stage extensive proliferation, TCR rearrangement and commitment to the αβ or γδ T cell lineages were described to happen (20). Our data, pointing the DN subset as strongly affected in the absence of galectin-3, might be related to critical interactions of the lectin with its ligands within specific niches in the subcapsular or cortical zones of the thymus. Further mechanistic studies are warranted to elucidate such a possibility.

It is also important to mention that in Gal-3^−/−^ mice, thymocyte death was increased in DP subset, the most numerous of the thymocyte subpopulations. Different data in the literature showed that galectin-3, a multifunctional molecule included in the class of matricellular proteins (1, 24, 25), acts both extracellularly, where it participates in cell adhesion and migration, and intracellularly, being able to regulate proliferation, apoptosis and cell signaling (3). In fact, galectin-3 was shown to protect cells from apoptosis, as it has the NWGR motif highly conserved in the BH1 domain of the Bcl-2 gene family, a well characterized suppressor of apoptosis, and was shown to interact with Bcl-2 (26, 27). Moreover, the expression of galectin-3 was shown to be upregulated in proliferating fibroblasts, suggesting a possible role for this lectin in the regulation of cell growth (28). Considering the important thymus atrophy noted in Gal-3^−/−^ mice, and that immature thymocytes are extremely sensitive to glucocorticoids we evaluated the levels of serum and thymus GC in these mice. Our results showed that Gal-3^−/−^ mice present extremely higher levels of serum and thymus corticosterone compared to WT. Previous works described the endocrine and paracrine actions of GC on thymocyte physiology, and that thymus presents the steroidogenic machinery, including StAR, 11β-HSD1 and 11β-HSD2 and is able to produce GC itself (15, 16), and express GC receptors (29, 30). Indeed, GC were shown to decrease proliferation and increase apoptosis of immature thymocytes (13, 14). Furthermore, blockage of GC receptors was shown to partially revert thymus atrophy observed in *Trypanosoma cruzi* infected mice (31). We showed here that the production of GC seems to be modulated by the lack of galectin-3, with concomitant increase in steroidogenic machinery both in the adrenals and thymus. We believe that high GC content in Gal-3^−/−^ mice may be involved in thymus atrophy, namely increased immature thymocyte death and decreased proliferation. We must also keep in mind that the lack of galectin-3, an anti-apoptotic molecule, may also be involved in the increased thymocyte death observed in Gal-3^−/−^ mice. To elucidate how much of the changes on thymocyte cellularity are due to hormonal changes and how much are due to the lack of galectin-3, we treated thymocytes from WT mice *in vitro* with a galectin-3 inhibitor (GCS-100) or with dexamethasone, and evaluated cell susceptibility to apoptosis. GCS-100 was previously shown to induce apoptosis in different cell lines and to regulate susceptibility to cell death (21, 22, 32). We showed here that thymocytes treated with different doses of GCS-100 were more susceptible to cell death *in vitro*. Moreover, double-positive thymocytes were the most affected cells, as observed also after dexamethasone treatment. From our new data, we suggest that both the increased GC contents and the lack of galectin-3 are likely to contribute to thymus atrophy in Gal-3^−/−^ mice. Taking together our *in vivo* and *in vitro* results, it is important to point out the divergence regarding cell death susceptibility of double-positive subset in the absence of galectin-3. Further studies approaching *in vivo* treatment of thymocytes with GCS-100 inhibitor are needed to clarify this point. Another important issue to be considered is the production of other galectins by thymic epithelial cells described to induce apoptosis in thymocytes, namely galectins −1, −8 and −9 (33–36). It is possible that the lack of the anti-apoptotic galectin-3, together with the presence of the pro-apoptotic galectins −1, −8 and −9 would unbalance thymocyte homeostasis, favoring thymocytes to be more sensitive to pro-apoptotic effects of other galectins secreted by the thymus microenvironment and even to apoptotic factors such as GC.

We should also have in mind that the lack of galectin-3 during all lifespan of Gal-3^−/−^ mice may have pleiotropic effects, influencing different organs that in sequence may affect the thymus. In fact, the lack of galectin-3 was shown to cause changes in the bone marrow, an important contributor to thymus cellularity with the generation of T cell precursors. This tissue was shown to be drastically modified in Gal-3^−/−^ mice with reduced cell density (37). Alterations on B cell precursors were defined, but nothing was described for thymocyte precursors (38). A detailed study on T cell precursors of Gal-3^−/−^ mice is missing.

Interesting changes in the thymus microenvironment were also observed in Gal-3^−/−^ mice in relation to WT, such as the appearance of concentric structures similar to Hassall bodies, which are not commonly seen in young mouse thymuses. Hassall bodies are formed by TEC expressing high molecular weight cytokeratins, and represent advanced stages of TEC maturation (39). These alterations may be related to the high corticosterone contents observed in our mice, as GC hormones are able to induce senescence and changes in cytokeratin and ECM expression in TECs (40–42). The existence of a galectin-3/GC circuitry has not been well established up to now and demands further studies. It is not clear if the levels of GC hormones interfere with galectin-3 secretion and vice-versa. However, previous studies showed that mice submitted to stress or macrophages treated with GC have decreased galectin-3 expression (43–45).

Another intriguing alteration that called our attention in the thymus of Gal-3^−/−^ mice was the presence of TEC-free regions in the thymic cortex, that were not observed in WT mice. Similar data were described previously in the thymus of aged mice and could be restored by oral zinc supplementation, as this chemical element is fundamental to thymus homeostasis, influencing the production of the thymic hormone thymulin, as well as TEC and thymocyte development (46). Cortical TEC-free regions have also been described in the thymus of different lupus strains of mice (NZB, MRL/MP-lpr/lpr, BXSB/MpJ Yaa, and C3H/HeJ-gld/gld), which undergo premature involution, but not in normal strains, including BALB/c, C57BL/6, and DBA (47). Changes in the thymic microenvironment, such as the occurrence of cortical TEC-free areas, may be harmful for T cell maturation, including positive selection that is dependent on cortical TEC/thymocyte interactions, reflecting in the accumulation of immature thymocytes and decreased thymus cellularity. Although no data relate these “TEC-free” regions to dysregulated selective events in the thymus, it is possible that the disturbed TEC-thymocyte interactions observed in Gal-3^−/−^ mice contributes to the decrease in thymus cellularity.

## Conclusion

Our data suggest that the lack of galectin-3 unbalances the steroidogenic machinery homeostasis in both thymus and adrenal, leading to an increase in local and systemic GC production, which in turn contributes to thymus atrophy by increasing thymocyte apoptosis and reducing thymocyte proliferation and TEC function. Besides, the direct effects of galectin-3 absence, such as defective intrathymic thymocyte migration, impaired proliferation and increased susceptibility to apoptosis, must be considered in the scenario of thymus atrophy observed in Gal-3^−/−^mice.

## Author contributions

EO performed all experiments and analyses, participated in study design and manuscript writing, DS participated in flow cytometry and immunofluorescence studies and analyses, AL participated in radioimmunoassay and performed qPCR analyses, MR participated in histological analyses, TR participated in immunofluorescence and proliferation studies, RF-R performed GCS-100 experiments, LV-F performed immunofluorescence analysis, RT participated in radioimmunoassay, VC-A participated in data interpretation and wrote the manuscript, VC performed radioimmunoassay studies, participated in data interpretation and wrote the manuscript, DV-V designed the study, participated in data interpretation and wrote the manuscript. All authors contributed to manuscript revision, read and approved the submitted version.

### Conflict of interest statement

The authors declare that the research was conducted in the absence of any commercial or financial relationships that could be construed as a potential conflict of interest.
